# Mechanistic Understanding of Sieving Lithium Ions Using a Biobased Sorbent Technology for Sustainable Lithium Reclamation and Cleansing Brines

**DOI:** 10.1021/acsomega.3c09716

**Published:** 2024-05-07

**Authors:** Kelvin Adrah, Sheeba Dawood, Hemali Rathnayake

**Affiliations:** † Nanoscience Department, University of North Carolina Greensboro, Greensboro, North Carolina 27401, United States; ‡ Minerva Lithium, LLC, Greensboro,North Carolina 27401, United States

## Abstract

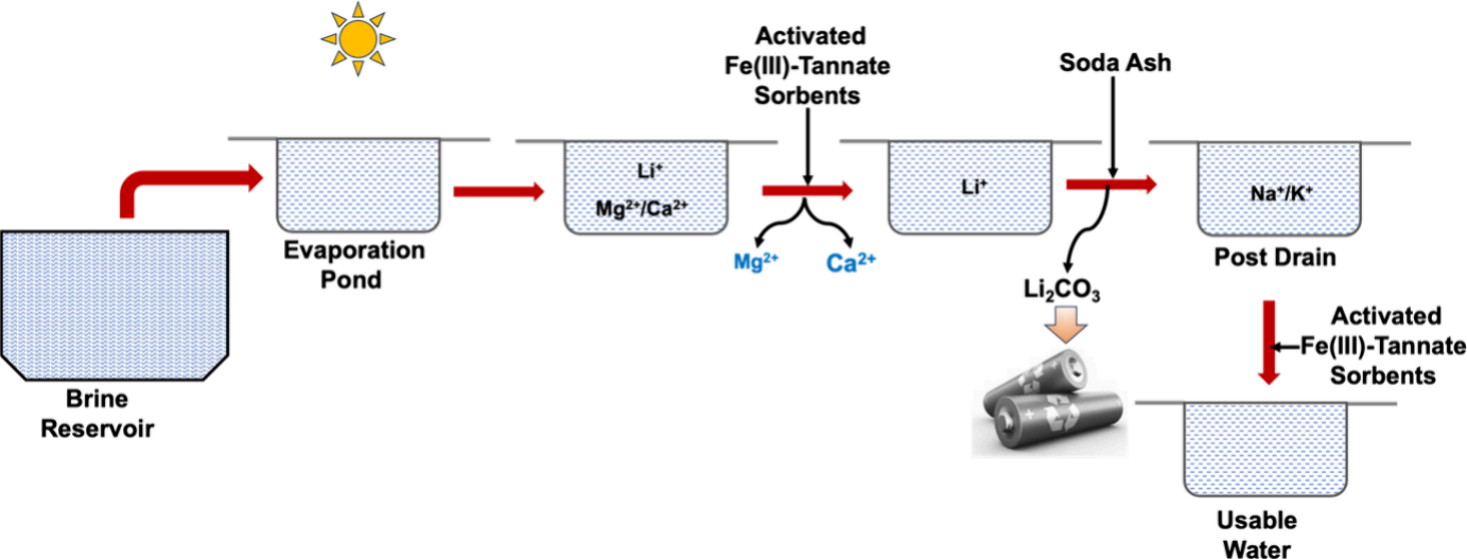

Low-cost environmentally benign materials that can be produced in a large scale to extract lithium from brine resources could drive the lithium market toward a clean technology with high lithium recovery and production. Herein, we have investigated the utilization of a novel, environmentally benign, and low-cost biobased sorbent for the extraction of lithium from lithium-rich solutions. This biobased molecular sieving sorbent, iron(III)-tannate (Fe(III)-TA), belongs to a novel class of coordination polymer frameworks derived from a natural polyphenol—tannic acid (TA)—coordinated with iron(III) metal cations. Its lithium adsorption and kinetic isotherm studies conducted using lithium-rich aqueous solutions confirm the sorbent’s dual function for lithium sieving via physisorption, chemisorption, and mass transfer diffusion processes. The adsorption equilibrium and kinetic isotherm models combined with the external and internal mass transfer diffusion models reveal a mechanistic pathway for lithium-ion adsorption. Aiding by forming a fluid film for external mass transfer diffusion of lithium ions, analytes adsorb onto the sorbent surface via physisorption and chemisorption followed by the internal mass transfer diffusion, occupying lithium ions in the sorbent’s pores. The lithium adsorption efficiency studies conducted for brines with different concentrations of interference alkali and alkaline cations evidence that the sorbent’s affinity for lithium ions strongly depends on the analyte concentration. The results evidence that the sorbent has the ability to lower the brine’s salinity and significantly reduces the ratios of Mg/Li and Ca/Li by 4-fold and 10-fold, respectively, yielding lithium-rich solutions. Thus, implementing this innovative biobased sorbent technology as an add-in step into traditional lithium extraction and refining processes, one can design a cost-effective pathway to yield lithium-rich leachate by reducing the Mg/Li and Ca/Li ratio. Nonetheless, the present work demonstrates that Fe(III)-tannate is an effective multifunctional sorbent for sieving lithium from lithium-rich aqueous solutions as well as for desalinating brine resources to recover usable water. Thus, this biobased sorbent offers the possibility of effective application of lithium reclamation and remediation of brine, mitigating the environmental impact of brine discharge and large volume of freshwater usage for lithium extraction and refining.

## Introduction

The use of lithium increases exponentially with lithium-ion battery production, which accounts an annual growth rate of 64%.^1^ Although global lithium reserves contain about 40.5 million tons of lithium, more than 85% of world’s recoverable lithium is in the liquid state.^2^ Thus, an innovative nanotechnology-enabled, simple, rapid, and low-cost lithium extraction method, which either removes interference ions, yielding a lithium-rich solution, or selectively sieves lithium, rejecting other ions from lithium-rich aqueous media, is appealing due to the ability of controlling the functionality, pore dimension, and selectivity at the molecular level. Low-cost environmentally benign materials that can be produced in a large scale to extract lithium from aqueous solutions are ideal materials to produce high-purity lithium. Nonetheless, compared to recovering lithium from hard-rock mining, recovery of lithium from brine is much cheaper, eco-friendly, and simple and drives the lithium market toward clean technologies.

For decades, commercial lithium production relies on mineral ores but is normally more expensive compared to recovery from brine resources.^1^ Brines from salars and salt lakes are the primary source of lithium, and geothermal brines represent secondary sources. Fracking produced brine from oil and gas operations is also currently an untapped source of lithium. Solar evaporation, chemical precipitation, adsorption with inorganic ion-exchange sorbents, solvent extraction, and concentration with membrane technologies are the primary methods of lithium recovery from brines.^3−5^ Among these lithium extraction technologies from brine or liquid solutions, ion exchange and solvent exchange are two technologies that have been promising as cost-effective lithium extraction methods. However, these two technologies are not adequate to produce high purity lithium in a large scale due to a low selectivity for lithium recovery (<65%) and degradation of ion-exchange columns.^3,4^ The lithium recovery using membrane technologies mainly depends on reverse osmosis (RO) and nanofiltration (NF). However, RO/NF is linked to the osmotic pressure, the membrane’s selectivity, and the mechanical stability of any associated filters. Conventional NF processes cannot efficiently separate lithium without heavy pretreatment of the brine, such as diluting the brine with a large amount of fresh water. The selectivity of membranes for monovalent ions over multivalent ions is the key factor in determining the efficiency of the recovery process. In contrast, the sorbent-based adsorption process presents a more sustainable and environmentally friendly alternative to conventional membrane approaches. In the sorbent-based adsorption, the phase transfer process occurs at the interface between a solid and an aqueous phase.^6,7^ In this process, the adsorbates adhere to the surface of a porous adsorbent through physical interactions, such as van der Waals forces and hydrogen bonding, or chemical bonding, or a combination of both mechanisms.^6,7^ The importance of sorbent-based adsorption is its reversibility between adsorption and desorption, particularly in the cases of physical adsorption, allowing for regeneration of the adsorbent. This regenerative capacity is pivotal for analyte extraction, separation, and water remediation processes.^7^ Toward this end, the most promising adsorbents for lithium recovery from aqueous solutions in the presence of interference ions are lithium manganese oxides (LMO).^8−10^ However, due to the chemical instability of the LMO sorbents during the desorption process by using hydrochloric acid, their economic performances in industrial settings are insufficient.

Moreover, the presence of magnesium and lithium in the concentrated mother brine is a significant challenge in current technologies, and an additional coprecipitation step is required to remove magnesium. Typically, the Mg/Li ratio in brine requires reducing below 6 to recover lithium economically by a chemical precipitation method.^8^ Unfortunately, the Mg/Li ratio in most brine resources is significantly high (×10–100), making it difficult to coprecipitate magnesium with a high recovery of lithium. Although there are many separation processes reported and some have been implemented on the commercial scale, their utilization in high Mg/Li brines remains as a scientific gap in sorbent-based separation methods, resulting in a challenge in terms of economic standpoint for the high-purity lithium recovery.^9−13^

Up to date, there are no efficient lithium extraction methods developed that can extract lithium by eliminating coprecipitation steps to remove interference ions, especially reducing the Mg/Li ratio in brine while desalinating high concentrations of total dissolved solids (TDS) to produce usable water. Overcoming these scientific gaps, herein, we aim to address the unmet need in the refining of lithium from brine resources by developing a rapid and cost-effective sorbent-based lithium recovery method. The novelty of this innovation over current lithium recovery technologies, such as chemical treatment in ore mining and solar evaporation and ion-exchange techniques in brine deposits, is the desalination and selective adsorption of divalent cations of magnesium and calcium via both surface adsorption and pore filling mechanisms. This study proves the reducing Mg/Li and Ca/Li ratios in brine, enabling the feasibility for rapid and efficient recovery of lithium within less than 2 days with ∼80% recovery of lithium-rich brines. Nonetheless, this study addresses the scientific gaps in the sorbent-based adsorption process of selective removal of divalent cations to yield lithium-rich brines and the recovery of usable water. The conventional sorbent-based methods, including ion-exchange techniques, uses large volumes of freshwater during the lithium recovery process and discharges untreated brines to the environment.^3−5^

Focusing on the in-depth adsorption studies of our previously developed novel biobased sorbent, iron(III)-tannate (Fe(III)-TA),^14^ for lithium, we reveal its feasibility as a platform for a sorbent-based method for lithium capture and refining from brine. The attractiveness of Fe(III)-TA is featured by its origin from a natural polyphenol, a readily available biomass byproduct derived from agricultural waste. Given that it has amphoteric nature, high-density functionality, and nanoporosity, this novel sorbent, prepared by coordination of an iron(III) metal cation onto polytopic tannic acid,^14^ aids tailoring the Li^+^ adsorption capacity. Employing equilibrium adsorption isotherm models, adsorption kinetic isotherms, and external and internal mass diffusion models, we report the proof-of-concept validation of the Fe(III)-TA sorbent’s lithium adsorption process, its mechanistic pathway, and separation of interference divalent cations, yielding lithium-rich brine. We utilized FTIR and XPS analysis to identify the compositional changes of the sorbents during the adsorption and desorption process, revealing the sorbent’s stability in a wide range of brine concentrations. The research results proved that the sorbent’s capability for dual function of analyte adsorption via interfacial surface charge interactions promotes chemisorption and physisorption following a pore-filling mechanism via an internal diffusion. Additionally, the case studies conducted using synthetic brine solutions with different concentrations of interference ions convey the utilization of this amphoteric sorbent. It imparts a dual benefit as our sorbent not only facilitates lithium sieving but also concurrently enhances the efficiency and cost-effectiveness of the methodology through its function for adsorption desalination and reduction of the Mg/Li ratio in the mother brine. The lessons learned from this work contribute to the first demonstration for a potential green lithium reclamation method, which operates at room temperature, using an environmentally benign sorbent, while cleansing brine to produce usable water.

## Experimental Methods

### Materials

Tannic acid (C_76_H_52_O_46_, molar mass = 1701.19 g mol^–1^), iron(II)acetate hexahydrate, 28% ammonium hydroxide, and anhydrous ethanol were obtained from Sigma-Aldrich. Unless otherwise stated, all chemicals were used as received. The sorbent, Fe(III)-tannate, was prepared in-house following the previous published procedure.^14^ Lithium standards were purchased from PerkinElmer, USA.

### Characterization

The chemical composition and functional groups were analyzed using Fourier transform infrared spectroscopy (FTIR-Varian 670-IR spectrometer). The morphology and the electron diffraction images of the sorbent were obtained from transmission electron microscopy (HR-TEM JEOL2100PLUS with STEM/EDS capability at 120 and 200 kV, respectively). Chemical oxidation states of each element were obtained by X-ray photon spectroscopy (XPS-Escalab Xi+-Thermo Scientific). Elemental compositions for Fe, C, and O were also obtained from XPS elemental survey analysis. Detection of lithium ions and other alkali and alkaline cations in brine solutions before and after being soaked with sorbents was analyzed using a simultaneous inductively coupled plasma optical emission spectrometer (Varian 710-ES ICP Spectrophotometer).

### Procedure for Surface Activation of Sorbents

The sorbents were activated using 2.8% NH_4_OH. In a typical activation process, the sorbents (10 g) were soaked in the prepared 2.8% NH_4_OH solution (20 mL) and sealed with paraffin film followed by reaching equilibrium for 24 h. The activated sorbents were recovered through vacuum filtration and dried under the hood overnight. During the activation process, there was no color change or sorbents’ degradation detected from the FTIR analysis. The pH levels of the activated sorbents in DI water were measured to be 7.58, compared to the pH of nonactivated sorbents (pH = 4.93) in DI water, confirming the deprotonation of the residual hydroxy groups on the sorbent’s surface.

### Adsorption Isotherm Batch Experiments

For adsorption equilibrium isotherm and kinetic studies, a fixed fitted glass column with an internal diameter of 1 in. was used. The column was tightly packed with activated sorbents (10.0 g). Adsorption equilibrium isotherm studies were performed for lithium solutions with initial concentrations of 250, 500, 750, 1000, 1250, 1500, and 2000 ppm. Each column was charged with the lithium solution (20 mL) by soaking for 24 h to reach equilibrium. The eluents were collected and analyzed for residual lithium ions using ICP-OES. Equilibrium kinetic studies were conducted for a lithium solution with a concentration of 250 ppm. An aliquot of 20 μL was taken out at various time intervals for 24 h and analyzed for residual lithium ions. The amount of lithium ions adsorbed onto the sorbent at equilibrium (*q*_e_) was calculated by mass balance equation of lithium-ion concentrations before and after adsorption using eq 1, as shown below:
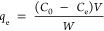
1where *q*_e_ is the equilibrium sorption capacity and *C*_0_ and *C*_e_ are initial and final lithium ion concentrations, respectively, at equilibrium.

### Desorption of Lithium from the Sorbents

Adsorbed lithium ions were stripped out from the sorbents, which were treated with the initial lithium-ion solution of 250 and 2000 ppm, by soaking the column in 35% acetic acid solution for 48 h. To do this, the treated sorbents were dried and redispersed into the glass column. Next, a 35% acetic acid solution (20.0 mL) was added and left undisturbed. At various time intervals (i.e., 3, 6, 12, 18, 24, and 48 h), an aliquot of 20 μL of acetic acid solution was taken out for ICP-OES analysis.

### Sample Preparation for Adsorption Analysis

Eluents were analyzed for lithium ions using a simultaneous inductively coupled plasma optical emission spectrometer (ICP-OES) with trace element analysis capability for wavelengths from 177 to 785 nm (Varian 710-ES ICP Spectrophotometer) in 3% (v/v) nitric acid solution. All measurements were taken in three replicates. In a typical sample preparation method for ICP analysis, the eluents were filtered through a 0.45 μm filter. Next, the filtered eluents were diluted to less than 100 mg/L concentration by adding 3% (v/v) nitric acid solution. For preparation of standards, the lithium standard (1.0 mL) was added to 3% (v/v) nitric acid solution (9.0 mL) to make a lithium stock standard with a concentration of 100 ppm. Using serial dilutions, other lower concentrations of the standards were prepared in 3% (v/v) nitric acid solution.

### Procedure for Zeta Potential Analysis of Lithium-ions Adsorbed Sorbents

For the zeta potential analysis, lithium solution (1000 ppm) was prepared by dissolving lithium sulfate (395.95 mg) in DI water (50.0 mL). Then, activated Fe (III)-TA (5.0 g) was soaked in lithium solution (10.0 mL). The suspension was allowed to sit undisturbed for 24 h. The adsorbents were collected by decanting the solution, and the recovered sorbents were dried in an oven for 4 h at 100 °C. The zeta potential of the sorbents with adsorbed lithium ions was measured using a Zeta sizer dip cell kit (Malvern Nano ZS, UK). The sorbents (20.0 mg) were dispersed in DI water (1.0 mL) and sonicated for 10 min to form a suspension. The pH of each solution was adjusted to make the pH of the suspensions 2, 4, 6, 8, and 10. The pH of each solution was adjusted by dropwise addition of 0.1 M HCl or 0.05 M NaOH. Three measurements of zeta potentials were taken for each sample.

### Procedure for Synthetic Brine Analysis

Synthetic brine solutions with different compositions of lithium ions and other interference alkali and alkaline cations were prepared by maintaining the ratio of Li^+^ concentration to alkali (Na^+^ and K^+^) and alkaline cations' (Mg^2+^ and Ca^2+^) concentrations at 1:1, 1:2, and 1:5. In a typical procedure for synthetic brine preparation, salts of respective cations with relevant weights were dissolved in deionized water (1 L). The lithium-ion concentration at 200 ppm was maintained by dissolving Li_2_SO_4_ (0.792 g) with other interference salts of NaCl (0.254, 0.508, and 1.27 g), MgCl_2_ as MgCl_2_6H_2_O (0.837, 1.673, and 4.183 g), CaCl_2_ as CaCl_2_2H_2_O (0.367, 0.734, and 1.834 g), and K_2_CO_3_ (0.177, 0.353, and 0.883 g) in deionized water.

## Results and Discussion

**Figure 1:**
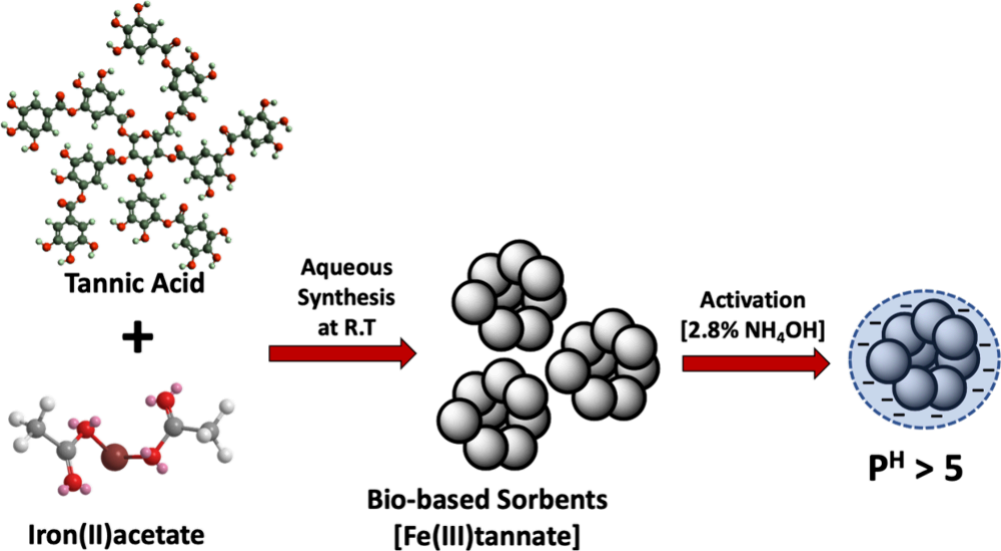
Schematic diagram representing the sorbent’s formation and its activation.

As the first bioinspired coordination polymer with a rigid framework synthesized from a polytopic natural polyphenol, the porous Fe(III)-TA sorbent demonstrates the use of biomass building blocks to construct an environmentally benign bioinspired adsorbent using a green synthesis in a large scale under ambient conditions in water (Figure 1).^14^ The structural and chemical composition of the sorbent was confirmed by FTIR spectroscopy (Figure S1a) and XPS elemental survey analysis (Figure S1b and Table S1). Microporosity, amphoteric surface properties, and colloidal stability in water at a wider range of pH as well as heavy metal adsorption highlight Fe(III)-TA’s potential utility as an amphoteric sorbent for separation, extraction, and upcycling of anions, cations, toxic heavy metals, valuable minerals, and organic contaminants from water resources.^14^ For instance, the physiochemical surface properties of this sorbent provides a greater insight into understanding the interfacial interactions with a variety of impurities present in aqueous solutions, enabling one to use it as an adsorbent for contaminant removal and separation from aqueous environments.^14^

Owing to the amphoteric nature of Fe(III)-TA sorbents, the solid-phase adsorption of Fe(III)-TA could offer a favorable path to extract one class of analytes over another. Favoring the cationic interactions with the sorbent’s surface, we conditioned the sorbents with a weak base to reach the pH of the colloidal solution above 5. As shown in Figure 1, sorbents were conditioned with 2.8% NH_4_OH solution to deprotonate hydroxyl groups within the catechol units of the sorbent, inducing a negatively charged surface. The increase in pH from 4.93 to 7.58 evidences the sorbent’s negatively charged surface upon activation. The FTIR spectrum of the activated sorbent (Figure S2) exhibits the retention of Fe(III)-O nodes in the coordination framework, confirming the chemical stability of the sorbents in 2.8% NH_4_OH solution. This tailored activation process enables the sorbent to selectively interact with alkali and alkaline cations present in brine while sieving the smallest cation, lithium ion, via the pore filling mechanism through the porous framework as well as rejecting all anionic impurities. We aim to prove this thesis by first performing the adsorption isotherm studies for lithium followed by investigating its potential utilization for sieving lithium ions from different compositions of brine solutions while reducing the salinity due to the presence of various other interferences alkali and alkaline cations.

### Adsorption Equilibrium Isotherms

For our comprehensive adsorption studies, we used a fixed-bed batch adsorption process employing a fitted glass column packed with activated sorbents in 2.8% NH_4_OH as described in the Experimental Methods. The effect of initial lithium-ion concentration on the adsorption capacity of the activated Fe(III)-TA sorbents was evaluated by fitting the adsorption isotherm data to Langmuir, Freundlich, and Temkin models.^15−17^ These three models are the most common isotherm models used for understanding the batch adsorption of the adsorbents. The Langmuir isotherm model assumes a monolayer adsorption process on a uniform adsorbent surface. The adsorbed molecules occupy a limited number of sites on the adsorbent surface, accommodating each active site by only one molecule. The general form of Langmuir isotherm model is represented by eq 2, and its linear form can be represented by eq 3. The Freundlich adsorption isotherm model conveys that the adsorbent surface is not homogeneous and that the energy and affinity also vary with the adsorption site.^16^ The model is applicable to the adsorption of solutes in solution and can be expressed by eq 4 and its linear form by eq 5:
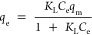
2
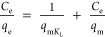
3
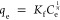
4
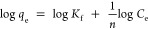
5where *q*_e_ (mg/g) is the equilibrium adsorption capacity, *q*_m_ (mg/g) is the maximum adsorption capacity, *C*_e_ (mg/L) is the concentration of lithium ions when the adsorption reaches equilibrium, *K*_L_ (L/mg) is the Langmuir equilibrium constant, *K*_f_ is the Freundlich isotherm constant [mg/g(L/mg)^1/*n*^], and *n* is the adsorption intensity.

The Temkin adsorption isotherm model considers the interaction between the adsorbent and adsorbate, regardless of the analyte concentration being high or low. However, the model is only valid for an intermediate concentration range.^17^ The linear form of the isotherm is expressed by eq 6:^17^
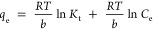
6where *K*_t_ is the equilibrium binding constant (L/g), which represents the maximum binding energy, *b* is the heat of adsorption (J/mol), *T* is the temperature in Kelvin, while *R* is the universal gas constant (8.314 J/K/mol). The plot of *q*_e_ vs In *C*_e_ produces a slope and an intercept for the derivation of *b* and *K*_t_, respectively.

**Figure 2:**
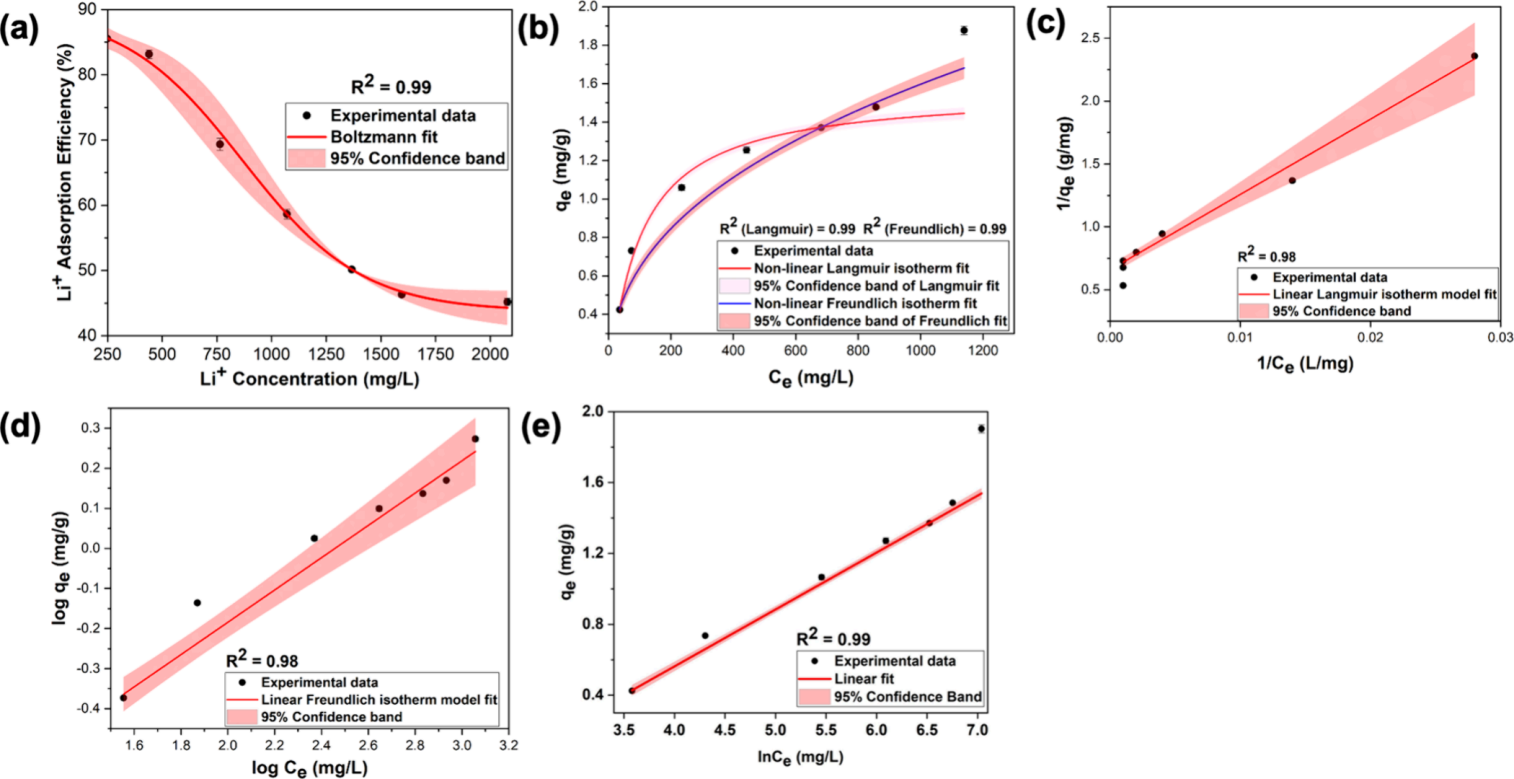
Equilibrium adsorption isotherms of lithium onto Fe(III)-TA. (a) % Adsorption of lithium ions with respect to the initial concentration of lithium ions at equilibrium. (b) Nonlinear curves of Langmuir and Freundlich isotherm models of lithium adsorption on the sorbent, generated from the experimental adsorption isotherm data. (c) Linear form of Langmuir isotherm, (d) linear form of Freundlich isotherm, and (e) Temkin isotherm.

The effect of initial lithium-ion concentration on the removal efficiency of the sorbents was investigated with respect to the initial lithium-ion concentration, ranging from 250 to 2000 ppm. As shown in Figure 2a, the percent adsorption decreases from the highest percentage removal of 85% at 250 ppm to the lowest percentage removal of ∼47% at 1500 ppm with no significant decrease in removal efficiency beyond this initial concentration. The result suggests that the adsorption of lithium ions onto the sorbent is concentration-dependent. The initial lithium-ion concentration affects the removal efficiency indirectly by either increasing or decreasing the availability of binding sites on the adsorbent. There is an immediate relationship between the percentage removal of adsorbate and the initial adsorbate concentration in batch adsorption systems.^18^ Generally, an increase in the initial adsorbate concentration in the solution causes the adsorption sites on the adsorbent surface to become saturated, which eventually leads to a decrease in the removal efficiency. Prior research studies evidence that the reduction of removal efficiency of adsorbate with respect to the increase in its concentration occurs normally with many kinds of adsorbents, where the mass transfer forces are low due to low initial concentrations of adsorbate molecules.^19,20^ Thus, as suggested by Albroomi et al.,^21^ it can be concluded that the occurrence in reduction of removal efficiency with respect to the initial concentration of lithium ions could be attributed to several factors, such as (1) at low initial lithium-ion concentrations, the availability of vacant pores and binding sites on the sorbent are high, where the fractional adsorption and mass transfer of lithium ions become high, leading to the higher percentage removals of lithium ions at the range of initial lithium-ion concentrations of 250 to 400 ppm, (2) as the initial lithium-ion concentrations increase beyond 400 ppm, the mass transfer force of lithium decreases, leading to gradual reduction in adsorption percentage onto the available binding sites of the sorbent, and (3) as the initial lithium-ion concentrations increases beyond 1500 ppm, the ratio of lithium ions to the available binding sites is at the optimal levels that do not support mass transfer, thereby saturating the adsorption at higher concentrations beyond the initial lithium-ion concentration of 1500 ppm.

To understand the adsorption performance of the sorbent for lithium ions, the adsorption equilibrium isotherm data was fitted into Langmuir, Freundlich, and Temkin isotherms and are shown in Figure 2b–d. Table 1 summarizes the adsorption equilibrium isotherm parameters calculated from all three isotherm models for the adsorption of lithium onto Fe(III)-TA sorbents. At equilibrium, the adsorption of lithium ions onto the sorbents agrees with the models based on a high correlation coefficient (*R*^2^) with a confidence level of above 98%. The equilibrium isotherm data, fitted to the nonlinear forms of the Langmuir isotherm model (Figure 2b), reveal a gradual increase in adsorption capacity at low lithium-ion concentrations (ranging from 50 to 400 ppm) followed by a stabilization of adsorption capacity at higher lithium-ion concentrations, representing the theoretical maximum amount of lithium ions that can be adsorbed onto the sorbent. The maximum adsorption capacity (*q*_max_) of the sorbents for lithium ions is determined to be 1.52 mg/g from the nonlinear Langmuir isotherm curve and is slightly lower than the experimental maximum adsorption capacity (1.87 mg/g). The well-fitted linear form of the Freundlich isotherm curve (Figure 2d) also suggests the occurrence of lithium-ion adsorption beyond the monolayer adsorption, where lithium-ion adsorption continues without reaching a specific maximum adsorption capacity. The linear forms of both isotherm models further convey that lithium-ion adsorption follow a combination of a monolayer chemisorption process and a multilayer physisorption process, evidencing that the sorbent consists with homogeneous and heterogeneous adsorption sites. As summarized in Table 1, the Langmuir equilibrium constant, *K*_L_, is calculated to be 0.01 L/mg and is related to the adsorption energy of active sites.^15^ A low value of *K*_L_ represents the low adsorption energy of active adsorption sites, evidencing a low affinity for the adsorbate. This further explain the low adsorption capacity of the sorbent for lithium ions and the adsorption of lithium ions is unfavorable at higher adsorbate concentrations.

**Table 1:** Isotherm Parameters of the Adsorption Equilibrium

adsorption isotherms							
Langmuir		Freundlich			Temkin		
K_L_ (L/mg)	*R*^2^	*K*_f_ (mg/g(L/mg)^1/*n*^)	*n*	*R*^2^	*K*_t_ (L/g)	*b* (J/mol)	*R*^2^
0.010	0.98	0.101	2.47	0.98	0.105	25.88	0.99

The equilibrium isotherm data fitted to the linear form of the Freundlich isotherm model also suggests heterogeneous adsorption where *K*_f_ relates the multilayer adsorption capacity with intensity of adsorption and *n* is related to heterogeneity of the sorbent.^22^ A relative *n* ≪ 1 indicates that the adsorption intensity is favorable over the entire range of concentrations studied, whereas *n* > 1 suggests that the adsorption intensity is only favorable at a specific range of high absorbate concentrations but much less at lower concentrations.^23^ From the linear Freundlich isotherms, *K*_f_ is calculated to be 0.101 (mg/g(L/mg)^1/*n*^) and *n* is found to be 2.47 from the slope of the linear isotherm in Figure 2d (Table 1). The very low value of *K*_f_ suggests poor adsorption capacity via multilayer adsorption, evidencing a less heterogeneity of the sorbent’s surface. The *n* value indicates that the adsorption intensity is favorable at high lithium-ion concentrations, promoting multilayer adsorption.

Following the linear form of the Temkin isotherm model, the coverage of the adsorbent surface increases with respect to the adsorbate concentration where the adsorption heat (Δ*H*, a function of temperature) of all molecules within the layer decreases linearly rather than logarithmically.^17^ The equilibrium binding constant (*K*_t_) is calculated to be <1, and the maximum binding energy (*b*) is positive, favoring an exothermic process for binding of lithium ions onto the adsorbent surface. The maximum binding energy is 7.713 kJ/mol and further suggests the favorable binding interactions between adsorbate’s active sites and the analyte. Overall, the Temkin model suits the adsorbent’s adsorption equilibrium for lithium-ion adsorption, where binding interactions are energetically rather favorable compared to the other two models, in which the adsorption is favored either at a very low concentration or very high concentration of analytes.

### Adsorption Kinetic Isotherms

Comparable to adsorption equilibrium isotherm models, linear or nonlinear modeling of the time-dependent adsorption of lithium ions onto the adsorbent can reveal the adsorption kinetics of the sorbent for lithium ions. Kinetic data of lithium-ion sorption was analyzed using pseudo-first-order, pseudo-second-order, and Elovich kinetic models. A pseudo-first-order model is used for a simple kinetic analysis of lithium-ion adsorption and can be expressed in its nonlinear form (eq 7)^23^ and linear form (eq 8),^24^ whereas a pseudo-second-order model is based on adsorption equilibrium capacity and can be represented in eqs 9 and 10.^24,25^ The pseudo-first-order model considers the rate of change that occurs in the uptake of adsorbate at reaction time to be directly proportional to the difference in the concentration and the rate at which the adsorbate is adsorbed with time:
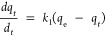
7
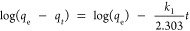
8where *k*_1_ is the rate constant for the pseudo-first-order adsorption (min^–1^), *q*_e_ is the adsorption capacity at equilibrium (mg/g), and *q_t_* is the adsorption capacity at time *t* (mg/g).
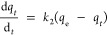
9
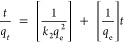
10where *k*_2_ is the rate constant of the pseudo-second-order adsorption.

The Elovich model describes chemical adsorption (chemical reaction) in nature and is typically suitable for systems with heterogeneous adsorption surfaces.^26^ Its nonlinear and linear forms can be expressed in eqs 11 and 12, respectively:^25^
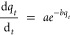
11
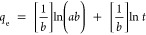
12where *a* and *b* are the initial adsorption rate (mg/(g min)) and desorption constant (g/mg), respectively.

**Figure 3:**
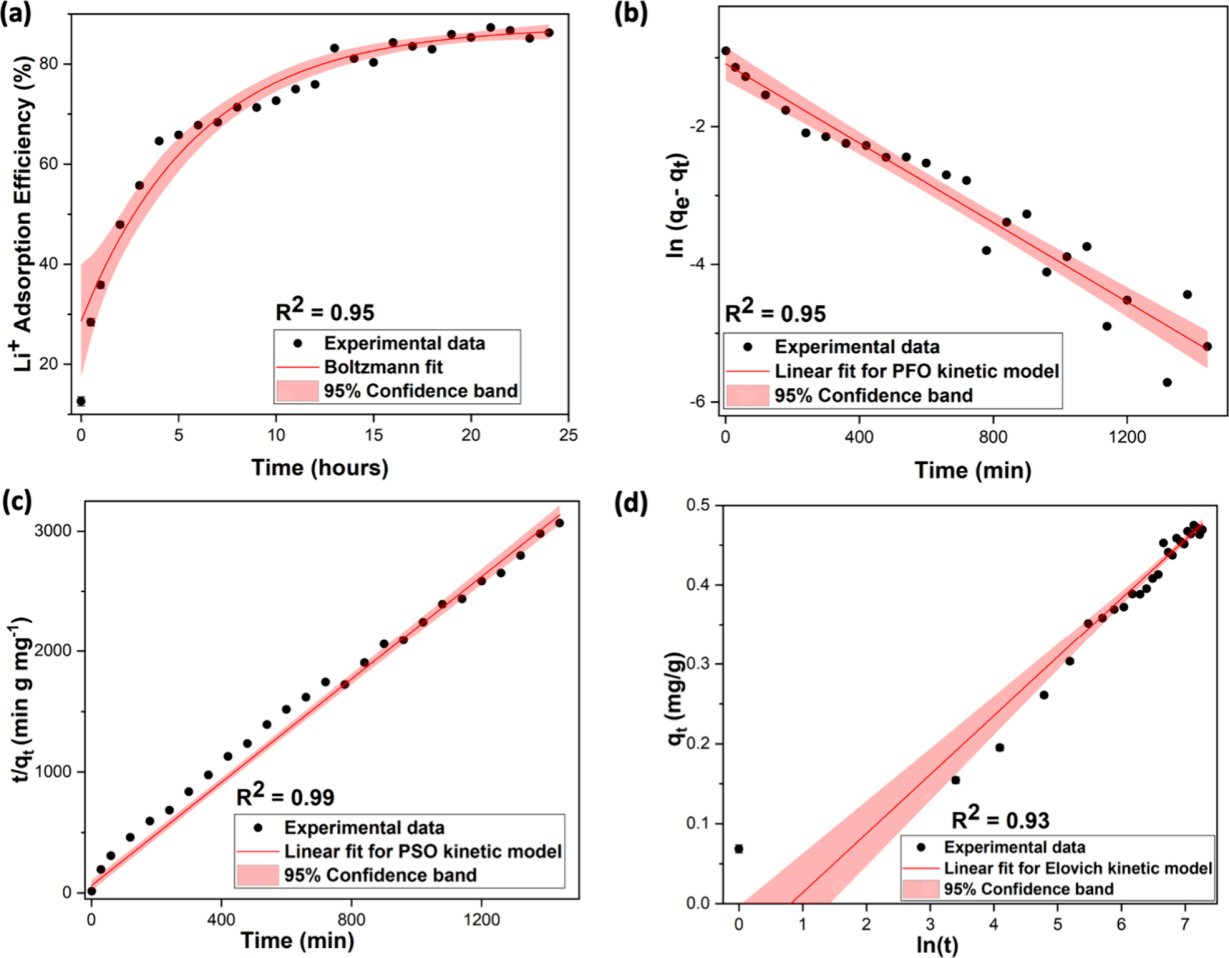
Adsorption kinetic isotherms: (a) % lithium adsorption with respect to the adsorption equilibrium time; (b) linear form of pseudo-first-order kinetic isotherm; (c) linear form of pseudo-second-order kinetic isotherm; and (d) linear form of Elovich kinetic isotherm.

The removal efficiency of Li^+^ by the sorbents, with respect to adsorption equilibrium time, shows a gradual increase in adsorption with time and then becomes saturated after 12.5 h, reaching a maximum lithium-ion removal efficiency of 88% at 24 h of adsorbate–adsorbent equilibrium (Figure 3a). The lithium-ion removal efficiency of above 88% at 24 h is comparable to the removal efficiency of lithium ions using metal oxide-based sorbents.^27^ The pseudo-first-order and pseudo-second-order kinetic isotherms revealed the nature of the adsorption mechanism of lithium onto Fe(III)-TA sorbents. As shown in Figure 3b,c, kinetic isotherm data fits very well to the pseudo-second-order kinetic model with a high correlation coefficient (*R*^2^) at a confidence level of 99% compared to the pseudo-first-order model, in which *R*^2^ is 0.95. As summarized in Table 2, the rate constant, *k*_2_, for the pseudo-second-order kinetic model is calculated to be 0.074 g/(mg min) and is larger than the pseudo-first-order kinetic model’s rate constant (*k*_1_), i.e., calculated to be 0.006 min^–1^.

**Table 2:** Parameters of Adsorption Kinetic Models

adsorption kinetic isotherms			
pseudo-first-order (min^–1^)		pseudo-second-order (g/(mg min)	
*k*_1_	*R*^2^	*k*_2_	*R*^2^
0.006	0.95	0.074	0.99

Based on both adsorption kinetic models and their respective rate constants (Table 2), it can be observed that the rate of Li-ion adsorption aligns with the pseudo-second-order kinetic model, which assumes that the rate-limiting step is chemisorption and predicts the adsorption behavior of Fe (III)-TA over the entire range of adsorbate concentrations. The experimental data, however, also fit with the pseudo-first-order kinetic model (*R*^2^ = 0.95). This indicates that the adsorption could also be susceptible to physisorption, reflecting the pseudo-first-order kinetic model. The correlation coefficient (*R*^2^) and the rate constant (*k*_2_) of the pseudo-second-order fitted model are greater than those of the pseudo-first-order fitted model (i.e., *k*_2_ > *k*_1_). The physical significance of these variables conveys the presence of both homogeneous and heterogeneous active surfaces on Fe(III)-TA sorbents, with a dominating chemical adsorption mechanism of lithium ions versus weak van der Waal forces, holding lithium ions on the adsorbent.

The kinetic data fitted into the linear form of the Elovich model also suggest lithium-ion adsorption via chemisorption at a higher equilibrium adsorption time, describing the second-order reaction kinetics for chemisorption onto an energetically heterogeneous surface (Figure 3d). The adsorption rate (*a*) is found to be 0.033 mg/(g min), and the desorption constant (*b*) is calculated to be 13.55 g/mg, which is significantly higher compared to most natural sorbents for cation adsorption, which follows the Elovich model.^26^ The low adsorption rate indicates that the multilayer chemisorption is slower than the pseudo-second-order kinetic rate, which promotes the chemisorption over the entire range of adsorbate concentrations. The high desorption constant reflects that the chemical interaction between the adsorbate and adsorbent is strong, hindering the desorption of lithium ions from the adsorbent’s surface. This limits further adsorption. Thus, the Elovich model conveys the insights into the kinetics of the process, and the values of the rate constant suggest strong chemical interactions and slow adsorption kinetics, making it challenging for the adsorbate to desorb from the surface.

### Diffusion Models

It is possible that lithium-ion adsorption may involve external and internal diffusion after adsorption onto the sorbent surface. If the sorption of lithium cations occurs on the sorbent surface, it may follow an external diffusion model. Owing to the porous nature of the sorbents, lithium cations could diffuse further into the pores internally. Thus, adsorption kinetic isotherm data was fitted to two diffusion models: an external diffusion model (eq 13)^28^ and an internal diffusion model, also known as the Weber and Morris model (eq 14).^29^ External diffusion can be explained by fitting the adsorption kinetic data into eq 13, which is represented as follows:
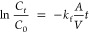
13
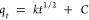
14where *C*_0_, *C_t_*, *A*/*V*, and *t* are the initial metal concentration, concentration at time *t*, external sorption area to the total solution volume, and sorption time, respectively. The external diffusion coefficient *k*_f_ (dm^3^/g min) can be calculated from the slope of the straight line obtained from eq 11. In eq 14, *k* is an internal diffusion coefficient (mg/g min^1/2^) and can be calculated from the slope of the linear curve, and *C* is the intercept that represents the boundary layer thickness (mg/g).

**Figure 4:**
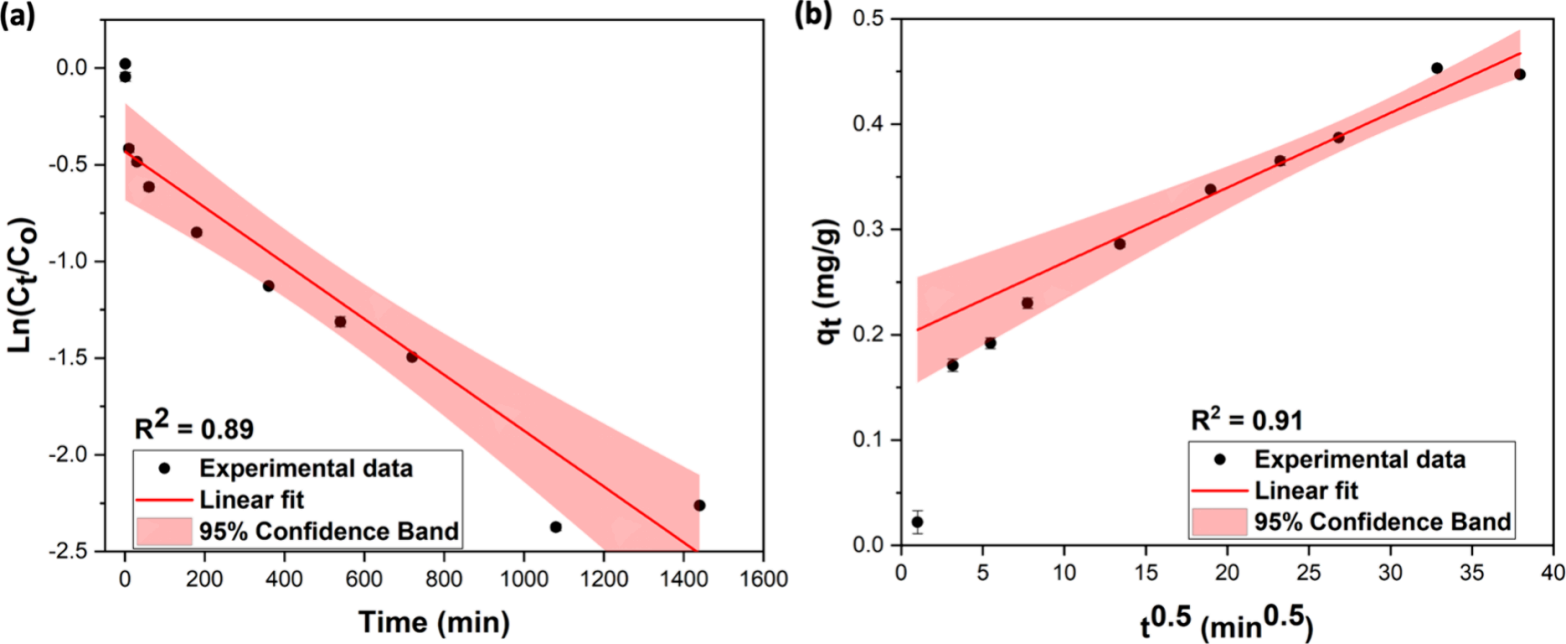
Plots of the (a) external diffusion model and (b) internal diffusion model for lithium cation adsorption.

The adsorption kinetic data fitted into the external diffusion model (eq 13) are shown in Figure 4a and exhibit some correlation with the sorption data obtained for the initial equilibrium time but deviate significantly beyond the equilibrium time of 600 min. This suggests that the adsorption of lithium ions is initially driven by the surface adsorption followed by external diffusion. The external diffusion coefficient (*k*_f_) was calculated to be 2.88 × 10^–6^ dm^3^/(g min), which is significantly smaller compared to the adsorption kinetic rate constants (*k*_1_ and *k*_2_ in Table 2), suggesting that the adsorption process is much faster than the external mass transfer diffusion of adsorbates. Thus, the external mass transfer diffusion of adsorbate onto the surface adsorption has some influence on the physical surface adsorption process, especially during the initial adsorbate–adsorbent equilibrium contact time.

The internal diffusion kinetic model based on the Weber–Morris equation (eq 14) is used to fit the adsorption data and is shown in Figure 4b. The plot is linear in nature with respect to the adsorption time and follows the internal diffusion model (eq 12) with 95% confidence (*R*^2^ = 0.91). Having an intercept, *C*, which is calculated to be 0.197 mg/g, indicates that that the rates of mass transfer in the initial and final steps of adsorption are different.^29^ Thus, the surface adsorption process of Li ions in Fe(III)-tannate sorbents involves both surface diffusion simultaneously along with intraparticle diffusion, contributing to the rate-controlling step. The internal diffusion coefficient (*k*) is calculated to be 0.007 mg/(g min^1/2^), which is comparable with the pseudo-first-order kinetic rate constant (*k*_1_) and one-tenth lower compared to the pseudo-second-order rate constant (*k*_*2*_). Thus, the internal mass transfer diffusion model suggests that the mass transfer of the adsorbate by diffusion into both the adsorbed solid phase and adsorbent’s pores occurs after the surface adsorption, following physical and chemical mechanisms. Overall, adsorption isotherm results and the mass transfer diffusion models evidence that Li^+^-ion sieving occurs via surface adsorption and external and internal mass transfer diffusion, saturating the sorbent’s surface-active sites and its pores.

### Li-Ion Adsorption Mechanism

**Figure 5:**
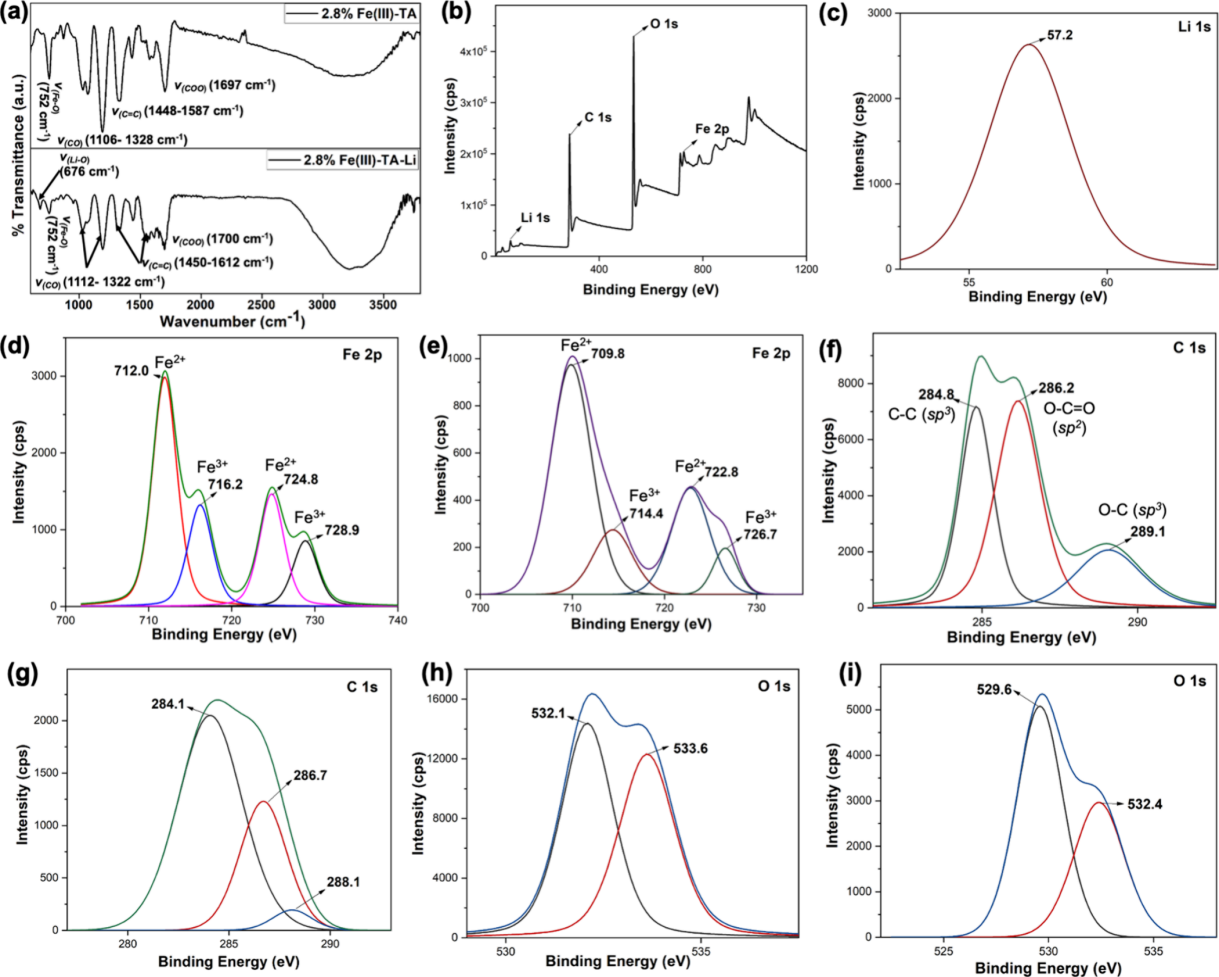
(a) FTIR spectral traces of activated Fe(III)-TA and Li^+^-bound Fe(III)-TA, (b) XPS elemental survey spectrum of Li^+^-bound Fe(III)-TA, and binding energy spectra of (c) Li 1s for Li^+^-bound Fe(III)-TA, (d) Fe 2p for Li^+^-bound Fe(III)-TA, (e) Fe 2p for activated Fe(III)-TA, (f) C 1s for Li^+^-bound Fe(III)-TA, (g) C 1s for activated Fe(III)-TA, (h) O 1s for Li^+^-bound Fe(III)-TA, and (i) O 1s for activated Fe(III)-TA.

To understand the nature of these interactions, we conducted FTIR and XPS analysis of the sorbent after soaking in lithium-ion solution (1000 ppm). The compared FTIR spectral traces are shown in Figure 5a. The presence of vibronic stretching at ν_(Fe–O)_ at 752 cm^–1^, ν_(C–O–C)_ at 1112 and 1322 cm^–1^, ν_(C=C)_ at 1450–1612 cm^–1^, and ν_(OC=O)_ at 1700 cm^–1^ in the FTIR spectrum of the Li^+^-bound Fe(III)-TA confirms the retention of the coordination framework.^14^ However, the noticeable shifts in ν_(C–O–C)_ and ν_(OC=O)_ evidence the interactions of carboxylate and ether oxygens with lithium ions. The appearance of an additional vibronic frequency peak at 676 cm^–1^, representing the ν_(Li–O)_ stretching, further supports the bonding interaction of Li ions to oxygen atoms in tannic acid’s catecholate hydroxyls and pyrogallol’s carboxylate groups. We observed a significantly broader vibronic stretching in the region of 330 cm^–1^ for soaked sorbents due to the adsorbed water molecules onto the sorbent’s surface.

Aligned with FTIR traces, XPS elemental survey spectrum (Figure 5b) and the binding energy spectrum of Li 1s (Figure 5c) confirm the presence of adsorbed Li ions on the sorbent’s surface and the retention of the coordination framework of Fe(III)-TA. The binding energy spectrum of Fe 2p for lithium-ion adsorbed Fe(III)-TA exhibits ∼2–3 eV shifts in binding energies (Figure 5d) compared to the binding energies of Fe 2p of activated sorbent (Figure 5e), confirming the chemical environment changes due to the adsorbent’s active site interactions with lithium ions. The well-resolved satellite binding energy peaks at 716.2 and 728.9 eV, which correspond to Fe 2p of Fe^3+^ oxidation states, in Li^+^ adsorbed Fe-TA further support the interference of Li-ion interactions with the coordination framework. The Fe 2p satellite binding energy peaks in activated Fe(III)-TA represent the Fe^3+^ oxidation state, which agrees with the uninterrupted chemical environment of the coordination framework. Aligned with the presence of ν_(Li–O)_ vibronic stretching in the FTIR spectrum of Li^+^-bound Fe(III)-TA, the binding interactions of Li^+^ to pyrogallol’s carboxylate (O–C=O, sp^2^) and ether oxygens (C–O, sp^3^) and catecholate’s hydroxyl oxygens (C–O, sp^3^) confirm from the respective well-resolved binding energy peaks at 284.8, 286.4, and 289.1 eV in C 1s spectrum of Li^+^-bound Fe(III)-TA (Figure 5f), compared to the binding energy spectrum of C 1s for activated Fe(III)-TA (Figure 5g). Similarly, O 1s binding energy spectral trace of Li^+^-bound Fe(III)-TA (Figure 5h,i) reflects the slight changes in the chemical environment from its binding energy shits due to the interference of adsorbed Li^+^ ions onto the carboxylate oxygens.

**Figure 6:**
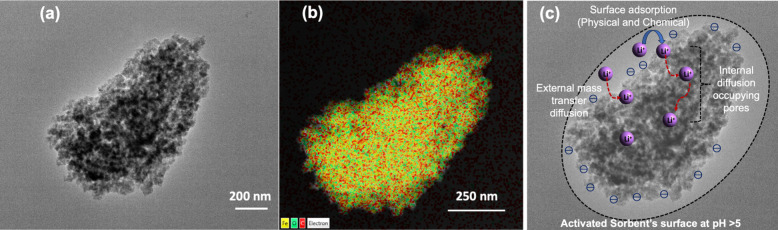
(a) HR-TEM image of a porous Fe(III)-tannate microstructure; (b) its respective electron diffraction image of the elemental map showing the distribution of Fe(III) metal ions; and (c) postulated Li^+^ sieving pathway based on the adsorption isotherm models and diffusion models.

The TEM image obtained for the microstructures of Fe(III)-TA exhibits a highly porous structure, as shown in Figure 6a. Its respective electron diffraction image depicted in Figure 6b reveals the distribution of coordinated metal-ion nodes within the coordination framework of Fe(III)-TA. Based on the adsorption equilibrium and adsorption kinetic isotherms, combined with the diffusion models, we could postulate that Li^+^ sieving occurs through cooperative participation of both surface adsorption and mass transfer diffusion of adsorbate. The chemical environment changes observed from FTIR and XPS analyses support the analyte interactions with the active sites of the sorbents. Thus, we can postulate that the adsorption mechanism follows the formation of the fluid film on the adsorbent’s surface via the external mass transfer diffusion, while the surface adsorption of Li^+^ occurs onto the sorbent’s active sites via physisorption and chemisorption. When the surface adsorption reaches its saturation, the internal mass transfer diffusion takes place, occupying the pores.

We postulate that our mechanistic pathway of Fe(III)-TA for adsorbing Li^+^ follows a stepwise process as illustrated in Figure 6c and includes the following steps: Step 1: forming a fluid film for external mass transfer diffusion where *k*_f_ ≪ *k*_1_ and *k*_2_; Step 2: surface adsorption of the analyte onto the active sites of the sorbent’s surface via both physisorption (Langmuir and Freundlich models) and chemisorption (Elovich model) following the pseudo-second-order kinetic model; and Step 3: occupying the pores via internal mass transfer diffusion where *k* ≅ *k*_1_ and *k* = [*k*_2_/10]. Therefore, we could conclude that Fe(III)-tannate sorbents extract lithium ions via both surface adsorption and pore filling mechanisms, offering an efficient method for sieving lithium from water resources. However, it is crucial to study the desorption process as the sorbent’s Li^+^-ion sieving involves both surface adsorption and pore filling by internal diffusion.

### Desorption Studies

**Figure 7:**
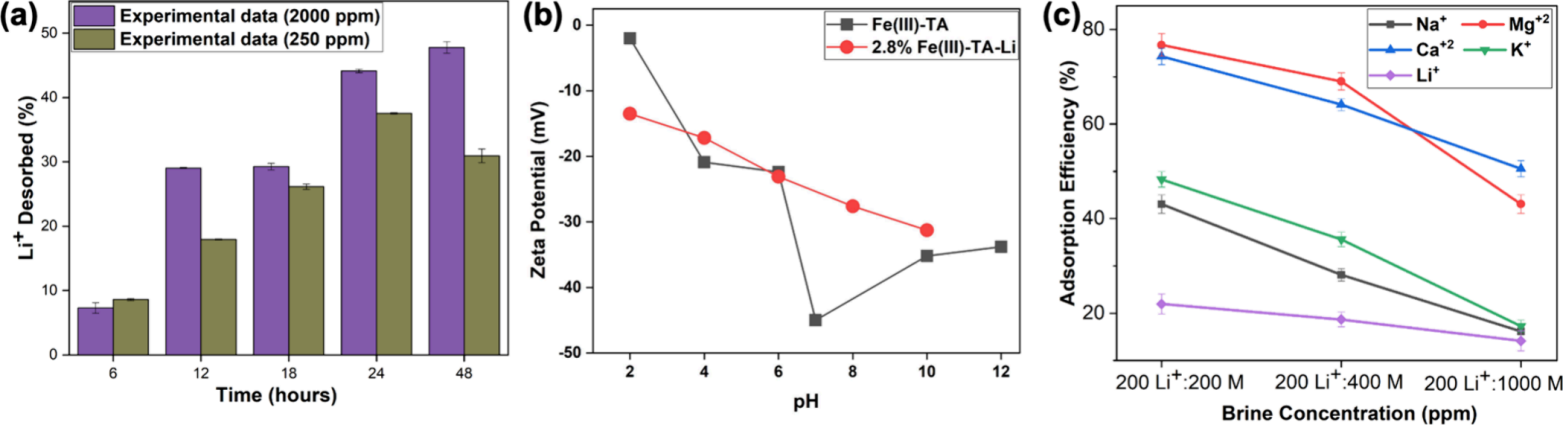
(a) Time-dependent %Li^+^ desorption plot for the 1st cycle of desorption using 35% acetic acid, (b) zeta potential vs pH plots for sorbents with adsorbed lithium ions and pristine sorbents, and (c) % adsorption of the activated sorbent using 2.8% NH_4_OH for alkali and alkaline cations in synthetic brines prepared with respect to different concentration ratios of Li^+^ to other interference alkali and alkaline cations.

Lithium-ion desorption studies were conducted for synthetic lithium solutions with initial concentrations of 250 and 2000 ppm. As our sorbent is a biobased sorbent, we used 35% acetic acid solution for stripping Li^+^ ions from the sorbents. The sorbents soaked in 250 and 2000 ppm solutions for 24 h were subjected to soaking in the acetic acid solution at 6 h time interval for 48 h. The time-dependent percent desorption for the first desorption cycle is shown in Figure 7a. It is revealed that the desorption capacity increases with time, but maximum desorption capacities after 48 h were calculated to be 30 and 48% for lithium solution initial concentrations of 250 and 2000 ppm, respectively. The low desorption efficiency indicates that attraction forces between the analyte and the sorbent are strong, and a weak acid, like acetic acid, is insufficient to strip most Li^+^ ions, which are chemisorbed onto the sorbent’s surface as well as occupied within the pores.

To understand the colloidal stability and the ionic strength of the sorbents with adsorbed lithium, we conducted pH-dependent zeta potential measurements for the used sorbents and compared with pristine sorbents in water. As shown in Figure 7b, sorbents with adsorbed lithium ions exhibit a gradual increase in colloidal stability with respect to pH. Additionally, compared to the zeta potential of pristine sorbents at pH 2, sorbents with adsorbed lithium ions exhibit considerably higher zeta potential, suggesting a rather stable colloids in the solution, indicating a higher ionic strength. The higher zeta potential for the used sorbents infers that sorbents with adsorbed lithium ions are prone to stay in the colloidal form, compared to the pristine sorbents at pH 2. This further suggests that desorption is less favorable. At higher pH, the desorption becomes less and less favorable for the sorbents with adsorbed lithium ions. Since we used 35% acetic acid, which has a pH of ∼4.5, the desorption is less favorable, yielding low desorption efficiencies.

**Figure 8:**
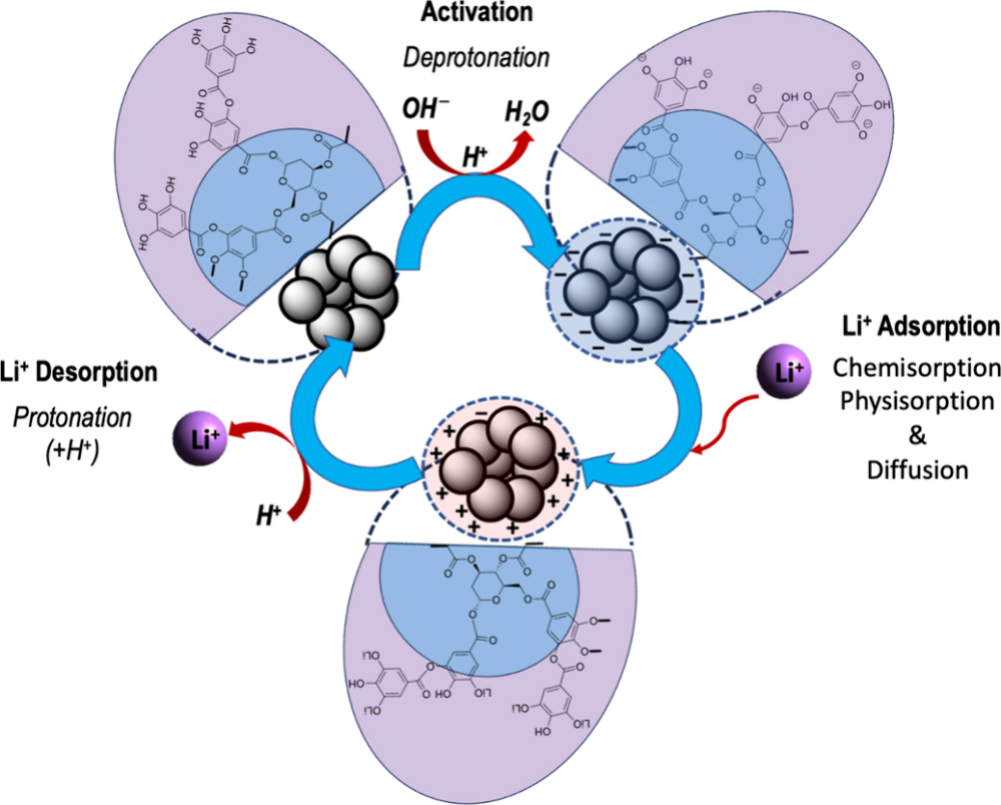
Schematic representation of the chemistry of the used sorbent regeneration process.

Owing to the colloidal instability of pristine sorbents and used sorbents in highly acidic solution (pH < 2), the desorption studies were not conducted using strong acids, like HCl, H_2_SO_4_, and H_3_PO_4_ acids. Instead, we performed repetitive desorption cycles in 35% acetic acid and were able to recover up to 61% after the fourth desorption cycle. However, we found that the adsorption efficiency and the maximum adsorption capacity were significantly decreased from 85 to 43% and from 1.87 to 0.48 mg/g, respectively, after the fourth desorption cycle of the sorbent for the initial lithium-ion concentration of 250 ppm (Table S3). Each regenerative cycle was performed by soaking the acetic acid-treated sorbents in DI water for 24 h followed by drying in a vacuum oven and finally activating with 2.8% NH_4_OH solution. This activation process of regenerating the sorbents follows the stepwise chemical process, in which the first step is stripping off lithium ions from the sorbents by exchanging lithium ions with acidic protons of acetic acid and then the second step is washing off physiosorbed lithium ions and acetic acids from the sorbent’s surface. The final step is activating the active sites of the sorbents by deprotonating the hydroxyl group of catecholate moieties using 2.8% NH_4_OH to yield a negatively charged surface. The schematic representation for our used sorbent’s regenerative process is depicted in Figure 8.

### Li^+^ Adsorption Studies Using Synthetic Brine Solutions

Lithium-ion adsorption efficiency of the sorbent was studied in the presence of other competing cations, such as Na^+^, K^+^, Mg^2+^, Ca^2+^, Al^3+^, and Mn^2+^, which are the most abundant interference cations in geothermal brines. The adsorption efficiencies of the sorbent toward Li^+^ and other cations, after 24 h of soaking in a fixed-bed column of activated sorbents, using 2.8%NH_4_OH are shown in Figure 6b–e. The Li^+^-ion adsorption in the synthetic brine solutions, with 1:1, 1:2, and 1:5 ratios of Li^+^ to competing ion concentrations, exhibits a significant reduction of the sorbent’s affinity for Li^+^ ions at all three different ion concentrations compared to the adsorption efficiency of Li^+^ ions without any competing ions (Figure 6b). On the other hand, sorbents favor adsorbing Mg^2+^ and Ca^2+^ with the adsorption efficiency ranging from 76 to 74% at 1:1 concentration of cations and maintain their adsorption efficiency at 43–50% even at higher concentrations of all other competing cations. In natural brines from Salt Lake resources, after the pretreatment to remove salts, the Mg/Li ratio is as high as 6:1, making the separation of lithium from magnesium by chemical precipitation difficult.^1^ The traditional chemical precipitation methods use soda ash to remove magnesium. Brines with a high Mg/Li ratio consume a higher amount of soda ash and coprecipitate lithium, resulting in a low yield of lithium.^1^ In our case, results show that our sorbents reduce both Mg/Li and Ca/Li ratios by one-third for all three different ratios, suggesting successful use of the sorbents for removing magnesium and calcium from brine and cutting down the cost for chemical precipitation. Thus, we dive into investigating the efficacy of sorbents for reducing the Mg/Li ratio in brine by testing two different compositions of brine (brines A and B) like the concentrated Salt Lake brines, with a low level of Na and K, and a brine with a very high level of NaCl (Brine C). Table 3 summarizes their respective adsorption efficiencies along with Mg/Li and Ca/Li ratios before and after treatment with the sorbents.

**Table 3:** % Adsorption Efficiencies of the Sorbents for Lithium and Other Interference Cations Present in Three Different Brine Samples along with Reduction Efficiencies of Mg/Li and Ca/Li Ratios

	brine A		brine B		brine C	
analyte	composition (ppm)	adsorption efficiency (%) ± SD	composition (ppm)	adsorption efficiency (%) ± SD	composition (ppm)	adsorption efficiency (%) ± SD
Li	77.13	33.93 ± 0.29	32.42	83.48 ± 2.70	66.02	20.07 ± 0.44
Na	1057.25	39.19 ± 0.21	2121.34	35.52 ± 0.86	18232.81	29.79 ± 0.10
Mg	1012.00	80.59 ± 0.08	2045.33	90.50 ± 0.26	139.61	25.34 ± 0.82
K	976.05	68.37 ± 0.11	1959.96	58.57 ± 0.66	407.68	58.69 ± 0.70
Ca	978.70	99.25 ± 0.05	1993.59	99.94 ± 0.03	665.46	85.29 ± 0.72

Mg/Li and Ca/Li concentration ratio in brine before and after adsorption						
	composition (ppm)	adsorption efficiency (%) ± SD	composition (ppm)	adsorption efficiency (%) ± SD	composition (ppm)	adsorption efficiency (%) ± SD
Mg/Li	13.12	3.85	63.09	36.29	2.11	1.98
Ca/Li	12.68	0.14	61.50	0.22	10.08	1.85

In brine sample A, the initial Mg/Li and Ca/Li ratios are ∼13 and 12 and are reduced to ∼4 and 0.1, respectively (Table 3). Notably, when the Li^+^ concentration is halved and the concentrations of Mg and Ca ions are doubled (brine B), the Mg/Li ratio decreases only by half. In contrast, the reduction of Ca/Li ratio was substantial and almost reduced by 280-fold. However, when the concentration of sodium is significantly high, the sorbent’s adsorption affinity is switched toward calcium ions the most, while exhibiting low adsorption efficiencies for lithium, magnesium, and sodium. Although further research is needed to make definitive conclusions, the initial results obtained from these brine samples, with a large variation in brine composition, indicate that the sorbent’s affinity for Li^+^ and the reduction of Mg/Li ratio strongly depend on the brine composition. Thus, our current and future research aim at understanding the equilibrium adsorption of the sorbents for Mg and Ca in the presence of lithium ions to demonstrate the sorbent’s ability for regulating Mg/Li and Ca/Li ratios. On the other hand, regardless of the heterogeneity of the TDS composition in brines, the removal efficiency of the sorbent toward calcium ions remains favorable. Therefore, the utility of the sorbent for reducing both Mg/Li and Ca/Li ratios is appealing and could be used as an add-in step for traditional lithium recovery methods to eliminate the coprecipitation of magnesium and calcium. In traditional methods, removing magnesium and calcium is a multistep process, which involves first treating with lime followed by soda ash and eventually treating with oxalic acid.^30^ Through this process, the recovery of lithium is less than 35%.^30^

## Conclusions

In summary, the present work on the proof-of-concept study for the establishment of a biobased sorbent technology for lithium reclamation demonstrates the potential utility of Fe(III)-tannate as a biobased sorbent for lithium recovery from lithium-rich brine resources. The sorbents exhibit a significant reduction of Mg/Li and Ca/Li ratios as well as salinity, resulting in lithium-rich leachate. The initial results on understanding lithium-ion adsorption equilibrium and kinetic isotherms of the sorbent demonstrate that the sorbent possesses high binding affinity for lithium with a lithium-ion absorption efficiency of ∼88% for aqueous solutions with lithium concentrations below 400 ppm. The Langmuir adsorption isotherm model applied for the adsorption equilibrium data conveys that the monolayer adsorption is favored at low lithium ion concentrations, yielding the maximum theoretical adsorption capacity (*q*_e_) of 1.52 mg/g. The Langmuir equilibrium constant, *K*_L_, was calculated to be 0.01L/mg, suggesting a low adsorption affinity for lithium. Compared to the Langmuir isotherm model, the equilibrium isotherm data fitted to the linear form of Freundlich isotherm model conveys the favorable adsorption of lithium ions via heterogeneous adsorption at the high-end range of adsorbate concentrations but unfavorable for lower concentrations of adsorbate. The intensity of adsorption, *n* ≫1, supports the favorable adsorption of lithium ions at high concentrations, promoting multilayer adsorption. However, considering the low binding constant (*K*_t_ < 1) and a considerably lower exothermic binding energy (b) obtained from the Temkin model, our sorbents favor the Temkin model for the adsorption of lithium ions. Thus, we can conclude that the adsorption equilibrium of the sorbents for lithium ions is in favor of following the Temkin model at the adsorbate concentration range of 50–1000 ppm.

The adsorption kinetic studies conducted by fitting the time-dependent adsorption equilibrium data for lithium ions confirm that the adsorbents follow the pseudo-second-order kinetic model, yielding a higher kinetic rate constant (*k*_2_ = 0.074 g/(mg min)) compared to the pseudo-first-order kinetic model, in which the rate constant was calculated to be 0.006 min^–1^. In summary, from the physical significance of rate constants, we can conclude that our sorbents possess both homogeneous and heterogeneous active surfaces, dominating the chemical adsorption mechanism for lithium ions over weak van der Waal forces of physisorption. The linear form of the Elovich model, which yielded an adsorption rate (*a*) of 0.033 mg/(g min), further confirms the lithium-ion adsorption via chemisorption at higher equilibrium adsorption times, agreeing with the pseudo-second-order kinetic model for chemisorption onto an energetically heterogeneous surface. The occurrence of external and internal mass transfer diffusion, confirmed by fitting the equilibrium kinetic isotherm data to two diffusion models, conveys the lithium-ion diffusion into the pores of the sorbents through the surface diffusion followed by internal diffusion of filling the pores. Thus, in summary, the adsorption isotherm models combined with mass transfer diffusion models have established the sorbent’s lithium-ion adsorption mechanism. It follows a stepwise process, consisting of an external mass transfer diffusion, followed by surface adsorption via physisorption and chemisorption, and an internal mass transfer diffusion, filling lithium ions into the pores.

The brine analysis results highlight the complexity of the selective sieving of lithium ions in the presence of alkali and alkaline cations in high concentrations. Although our results show that the sorbent’s affinity for lithium ions was significantly reduced with interference of other cations, its removal efficiency of Mg and Ca was remarkably higher, with 4-fold and 10-fold reduction of Mg/Li and Ca/Li ratios. Thus, one can utilize this biobased sorbent technology as an add-in step to eliminate the coprecipitation steps for removing Mg and Ca during the refining process in traditional lithium extraction and recovery methods, lowering the refining cost for chemical precipitation. Additionally, the sorbent’s potential for the desalination of brine at room temperature with minimal energy consumption and zero carbon emission could offer a path to cleanse brine for producing usable water, minimizing the freshwater usage for refining and mitigating the environmental impact due to the brine discharge into the groundwater and aquatic environment. Moreover, the application significance of our results will benefit brine operators, lithium producers, and water purification companies as our biobased sorbent technology reveals the scientific knowledge on its utility for solid-phase extraction processes to recover lithium from brine resources. As a result, the scientific insight presented herein with the following design pathways could enable accelerating the traditional lithium reclamation processes, with minimal environmental impact, and recovering usable water from brine resources.

The design pathways revealed from our results for using Fe(III)-tannate as a biobased sorbent technology by integrating into the traditional lithium extraction methods and lithium refining technologies are as follows:

1. Sequence the amphoteric nature of the sorbent’s surface-active sites to capture interference ions, initializing only the surface adsorption process in the first fixed-bed column as the adsorption mechanism follows a stepwise process.
2. Utilize the activated sorbents in a fixed-bed column to reduce Mg/Li and Ca/Li ratios at room temperature, eliminating the coprecipitation steps for removing Ca and Mg using lime, soda ash, and oxalic acid in a multistep process.
3. Utilize the sorbents to desalinate the brine to produce usable water by passing brine through a Fe(III)-tannate sorbent-filled column at room temperature, cutting down the energy usage and carbon emission.

Nonetheless, the design pathways could open the path to sorbent-based direct lithium extraction (DLE) and refining technologies, which would be the future driving force for overcoming the high demand for lithium with minimal environmental impact and zero carbon emission.

## Supporting Information Available

Adsorption and desorption results with respect to desorption cycles (FTIR spectra and XPS survey, elemental composition and binding energies, and adsorption and desorption efficiency and maximum adsorption capacity of the sorbents) (PDF)
