# Supplementary material for: Mechanistic Understanding of Sieving Lithium Ions Using a Biobased Sorbent Technology for Sustainable Lithium Reclamation and Cleansing Brines
Source: ACS Omega. 2024 May 7;9(20):21917–29. doi: 10.1021/acsomega.3c09716 (PMC11112576; doi:10.1021/acsomega.3c09716)
Supplement: Supplementary file 1 — ao3c09716_si_001.pdf [file ao3c09716_si_001.pdf]

Supporting Information

Mechanistic Understanding of Sieving Lithium Ions using a Bio-based Sorbent Technology for Sustainable Lithium Reclamation and Cleansing Brines

Kelvin Adrah,[1] Sheeba Dawood,[2] and Hemali Rathnayake.[1,]*

[1]Nanoscience Department, University of North Carolina Greensboro, Greensboro, NC, 27401, USA; [2]Minerva Lithium, LLC, Greensboro, NC 27401, USA

**Correspondence: hprathna@uncg.edu; Tel.: 01-336-285-2860.*

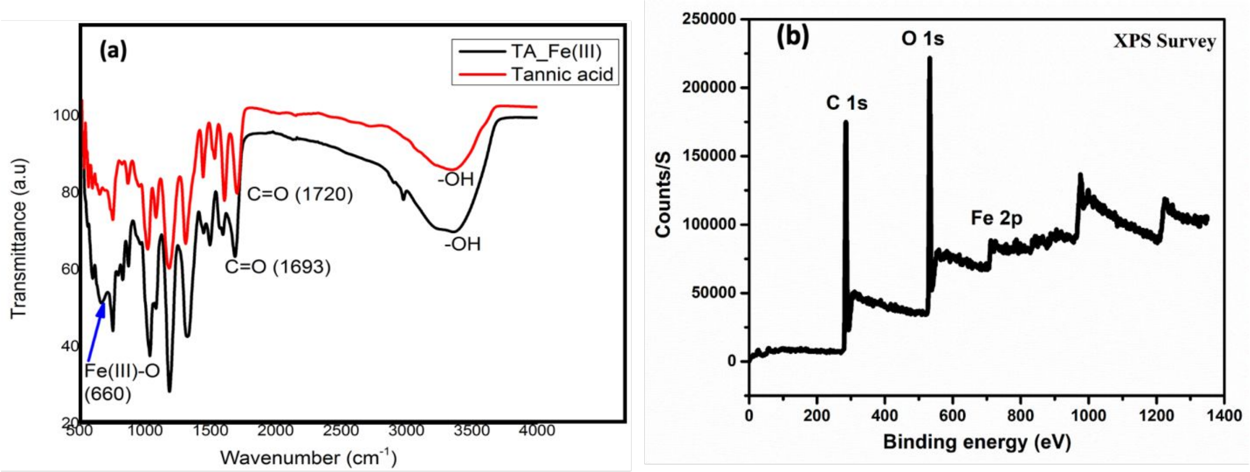

Figure S1: (a) FTIR spectra of Fe(III)-TA sorbents and tannic acid, and (b) XPS survey spectrum of Fe(III)-TA sorbents.

Table S1: Elemental composition and the binding energies of pristine Fe(III)-TA

Element type	%Elemental composition		Binding energies (eV)	Bonding type/oxidation state
	Experimental	Theoretical		
C 1s	47.25	46.28	284.1, 286.7, 288.1	C-C (*sp*^{3}), O-C=O (*sp*^{2})
O 1s	41.05	39.39	529.6, 531.0, 532.4	Fe-O, C-O (sp^3), C=O (sp^2)

Fe 2p	11.70	11.96	709.8, 714.4, 722.8, 726.7	$2p_{3/2}$ /Fe^{+3}, $2p_{3/2}$ /Fe^{+3} (Satellite Peak), $2p_{1/2}$ /Fe^{+2}, $2p_{1/2}$ /Fe^{+3}

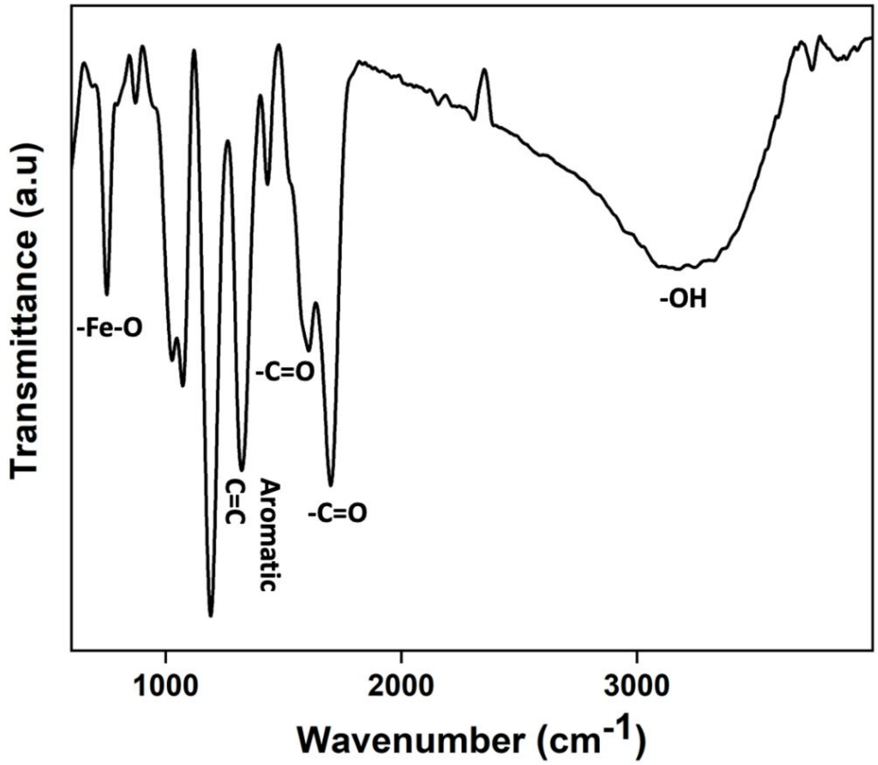

Figure S2: FTIR spectra of activated Fe(III)-TA sorbents.

Table S2. Elemental compositions and the binding energies obtained from the bulk analysis of XPS elemental survey for Fe-TA sorbents after lithium adsorbed.

Element type	**%Elemental composition**		**Binding energies (eV)**	**Bonding type/oxidation state**
	Experimental	Theoretical		
C 1s	39.16	46.28	284.8, 286.2, 289.2	C-C (sp^3), O-C=O (sp^2)
O 1s	32.03	39.39	532.1, 533.6	*Fe-O, C-O* (sp^3), *C=O* (sp^2)
Fe 2p	11.69	11.96`	712.0, 716.2, 724.8, 728.9	$2p_{3/2}$ /Fe^{+3}, $2p_{3/2}$ /Fe^{+3} *(Satellite Peak)*, $2p_{1/2}$ /Fe^{+2}, $2p_{1/2}$ /Fe^{+3}
Li 1S	17.12	-	57.2	Li^+

Table S3: Adsorption and Desorption efficiency and the maximum adsorption capacity of the sorbents with respect to four consecutive regenerative cycles.

# of Cycles	%Adsorption± 1.0	%Desorption	Adsorption capacity (mg/g)
1	82.8	21.8	1.87
2	42.5	49.2	1.00
3	41.9	48.3	0.48
4	43.9	61.0	0.48
